# Variability in the type and layer distribution of cortical Aβ pathology in familial Alzheimer’s disease

**DOI:** 10.1111/bpa.13009

**Published:** 2021-07-28

**Authors:** Nanet Willumsen, Teresa Poole, Jennifer M. Nicholas, Nick C. Fox, Natalie S. Ryan, Tammaryn Lashley

**Affiliations:** ^1^ The Queen Square Brain Bank for Neurological Disorders Department of Clinical and Movement Neurosciences UCL Queen Square Institute of Neurology London UK; ^2^ Department of Neurodegenerative Disease UCL Queen Square Institute of Neurology London UK; ^3^ Department of Medical Statistics London School of Hygiene & Tropical Medicine London UK; ^4^ Dementia Research Centre Department of Neurodegenerative Disease UCL Queen Square Institute of Neurology London UK; ^5^ UK Dementia Research Institute at University College London London UK

**Keywords:** Alzheimer's disease, amyloid, familial Alzheimer’s disease

## Abstract

Familial Alzheimer's disease (FAD) is caused by autosomal dominant mutations in the *PSEN1*, *PSEN2* or *APP* genes, giving rise to considerable clinical and pathological heterogeneity in FAD. Here we investigate variability in clinical data and the type and distribution of Aβ pathologies throughout the cortical layers of different FAD mutation cases. Brain tissue from 20 FAD cases [*PSEN1* pre‐codon 200 (n = 10), *PSEN1* post‐codon 200 (n = 6), *APP* (n = 4)] were investigated. Frontal cortex sections were stained immunohistochemically for Aβ, and Nissl to define the cortical layers. The frequency of different amyloid‐beta plaque types was graded for each cortical layer and the severity of cerebral amyloid angiopathy (CAA) was determined in cortical and leptomeningeal blood vessels. Comparisons were made between FAD mutations and *APOE4* status, with associations between pathology, clinical and genetic data investigated. In this cohort, possession of an *APOE4* allele was associated with increased disease duration but not with age at onset, after adjusting for mutation sub‐group and sex. We found Aβ pathology to be heterogeneous between cases although Aβ load was highest in cortical layer 3 for all mutation groups and a higher Aβ load was associated with *APOE4*. The *PSEN1* post‐codon 200 group had a higher Aβ load in lower cortical layers, with a small number of this group having increased cotton wool plaque pathology in lower layers. Cotton wool plaque frequency was positively associated with the severity of CAA in the whole cohort and in the *PSEN1* post‐codon 200 group. Carriers of the same *PSEN1* mutation can have differing patterns of Aβ deposition, potentially because of differences in risk factors. Our results highlight possible influences of *APOE4* genotype, and *PSEN1* mutation type on Aβ deposition, which may have effects on the clinical heterogeneity of FAD.

## Graphical abstract

An investigation of the frequency and distribution of the Aβ pathologies throughout the cortical layers of different familial Alzheimer's disease mutations cases. The frequency of different Aβ plaque types were graded for each cortical layer and the severity of cerebral amyloid angiopathy was determined in cortical and leptomeningeal blood vessels.

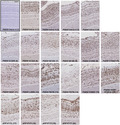

## 1 INTRODUCTION

Familial Alzheimer's disease (FAD) is caused by dominantly inherited mutations in *APP*, *PSEN1* and *PSEN2*. *APP* generates the amyloid precursor protein (APP), which is cleaved to produce the amyloid‐beta (Aβ) peptide. PSEN1/PSEN2 are the catalytic subunits of the ƴ‐secretase complex, which are involved in the production of Aβ (1, 2). As in sporadic AD, these Aβ peptides aggregate into extracellular deposits as amyloid plaques, a classic neuropathological hallmark of AD. Although the causative mutations all affect proteins required for Aβ production, considerable heterogeneity can be seen in the clinical and neuropathological features, which may indicate individual mutation effects (3, 4, 5).

Patients with FAD typically present with early amnestic symptoms, although atypical cognitive symptoms and additional neurological features are also seen (3, 6, 7). While FAD typically causes young onset dementia, with age at onset usually ranging between 30 and 60 years, late‐onset cases are increasingly found as older cohorts are sequenced (8, 9). In general, *PSEN1* mutations cause younger‐onset than *APP* mutations, while *PSEN2* mutations are associated with the oldest ages at onset (3, 9, 10). *PSEN1* mutation site has been found to influence age at onset, with mutations before codon 200 associated with a younger onset than those located beyond codon 200 (3, 11). Although mutation type accounts for a large proportion of the variance in age at onset, substantial variation can still be observed even within carriers of the same mutation and within families (3, 6, 9). Interestingly, despite its role as a risk factor for sporadic AD, *APOE* genotype was not found to significantly influence age at onset in a meta‐analysis of FAD (9). In contrast, the *APOE4* allele has been reported to be associated with younger ages at onset in a single large Colombian kindred harbouring the *PSEN1* E280A mutation (12). Other factors, which are relevant in sporadic AD, may also play a role in clinical aspects of FAD, although they are less well understood. For example, females have a higher risk of dementia overall compared to males and in a meta‐analysis of FAD, males showed a trend to a later age at onset (9).

Although symptoms tend to begin at a younger age when individuals’ general health is better, FAD generally gives rise to similar disease durations to sporadic AD (6, 9, 13, 14). However, this too can vary within FAD, with *PSEN2* cases reported to have longer disease durations than *PSEN1* and *APP* cases (10). No difference in duration was found between *APP* and *PSEN1* mutation carriers, nor between carriers of *PSEN1* mutations located pre‐ and post‐codon 200 (15). In a meta‐analysis, shorter disease durations were observed in those with younger (<35 years) or older (>65 years) onset than in those with onset in mid‐life (9). This same ‘inverted U‐shape’ association between age at onset and disease duration was found in a recent study from our centre when all mutations were considered together. However, it resolved when the different genetic groups were examined separately as later ages at onset were associated with longer disease durations in *PSEN1* mutation carriers, whereas later ages at onset were associated with shorter disease durations in the *APP* cohort. This study also found that survival in FAD was influenced by mutation to a much lesser extent than the age at onset and appeared to be longer in individuals with *PSEN1* mutations who carried an *APOE*4 allele (14).

In FAD, the Aβ isoforms produced and the relative ratios of these peptide species differ between pathogenic mutations (16, 17). A study of *APP* mutations has demonstrated that the distinct Aβ peptides produced by different mutations have distinct aggregation conformations and so influence the type of amyloid pathology produced, resulting in histological diversity between mutations (18). Post‐mortem analysis of Aβ pathology in FAD shows considerable heterogeneity between cases, with reports suggesting total Aβ may be more severe compared to patients with sporadic AD (19). The types of plaques found in the cortex are classified by their morphological features (20, 21) and within *PSEN1* cases the type and number of deposits show variability across different mutations (22). Additionally, between *PSEN1* pre and post‐codon 200 mutations, distinct histological phenotypes have been noted, with mutations post‐codon 200 tending to have more severe cerebral amyloid angiopathy (CAA) (11), which is the deposition of Aβ in blood vessel walls. Severe CAA has also been observed in cases with *APP* mutations located within the Aβ coding domain [reviewed in Ref. (4)]. These histological differences may be underlying causes of variable disease phenotypes.

As well as morphological differences between mutations, there is also variability in Aβ distribution across the depth of the cortex. In both AD and FAD, a consistent maximal density of Aβ pathology has been found in cortical layer 3, with varying densities in other layers (23, 24, 25, 26, 27). Additionally, Aβ plaque types have differing distribution patterns over the cortical layers (28, 29). However, a previous study examining a group of 20 AD patients, including four cases with FAD mutations, reported that Aβ density and plaque‐type distribution across the cortex did not appear markedly to differ between cases, although the cortical layers were not specifically delineated (30). Yet within FAD, certain plaque types have been associated with specific clinical features, such as the frequent observation of cotton wool plaques (CWP) in patients with spastic paraparesis (31, 32). These observations are particularly associated with certain mutations, such as *PSEN1* exon 9 deletions (33).

This study aimed to gain a deeper understanding of the association of genetic mutations to the morphology and distribution of Aβ pathology and their associations with disease pathogenesis in FAD. We analysed in detail the morphology and distribution of Aβ pathology, using cortical layer‐specific distributions, in a cohort of 20 FAD cases, using a range of measures and explored relationships with clinical data (age at onset and disease duration) and genotypes. Associations were examined for the cohort as a whole and also in exploratory analysis in the three mutation sub‐groups (*PSEN1* pre‐codon 200, *PSEN1* post‐codon 200 and *APP*).

Access to FAD post‐mortem tissue is limited and, as this was a study of cases donated to a single centre, our sample size was therefore small. This constrained our approaches when analysing the data and interpreting results, particularly for analysis in the mutation sub‐groups. In this context, while we report statistical significance for results in the whole cohort, our focus was on identifying trends and patterns. Our hypothesis was that a detailed examination of Aβ pathology in a variety of FAD mutations would reveal differences in Aβ plaque‐type and pattern of distribution across the cortical layers and in severity of CAA. We aimed to determine whether there are specific associations between the different types of Aβ pathology, and if these associations are observed in the FAD mutation‐specific sub‐groups.

## 2 MATERIALS AND METHODS

### 2.1 Cases

All 20 FAD cases were obtained through the brain donation program of the Queen Square Brain Bank for Neurological Disorders (QSBB), Department of Clinical and Movement Neurosciences, UCL Queen Square Institute of Neurology and all available FAD cases were investigated. The protocols used for brain donation and ethical approval for this study were approved by a London Research Ethics Committee and tissue is stored for research under a license from the Human Tissue Authority. The standard diagnostic criteria for the neuropathological diagnosis of AD and the presence of CAA were used in all cases (34, 35, 36, 37, 38).

### 2.2 Histological staining and Immunohistochemistry

Paraffin‐embedded serial sections (8 μm thick) were cut from the frontal cortex. For Nissl staining, deparaffinised and rehydrated sections were stained with 0.1% cresyl violet‐acetate (83860.120, Prolabo) to determine the cortical layers. Serial sections were used for Aβ immunohistochemistry (IHC), using a pan‐Aβ antibody that recognises all Aβ isoforms containing residues 8–17. Slides were pre‐treated in formic acid followed by pressure cooker in citrate buffer pH6.0. Endogenous peroxidase activity was blocked with 0.3% H_2_0_2_ in methanol and non‐specific binding with 10% dried milk solution. Sections were incubated with the primary Aβ antibody (1:100; M0872, DAKO) overnight at 4°C, followed by biotinylated anti‐mouse (1:200, 30 min; E0354, DAKO) and ABC complex (30 min; PK‐6100, Vector Laboratories Ltd). Colour was developed with di‐aminobenzidine/H_2_O_2_. Slides were scanned and digitised using a Leica slide scanner with a 40x objective.

### 2.3 Layer delineation

Digitised Nissl sections were used to delineate the cortical layers, the second frontal gyrus (Brodmann area 9) was used for analysis. Regions of analysis were used in Adobe® Photoshop® software (Adobe® Photoshop® CC 2017, Adobe Systems Incorporated) to delineate and mark the six cortical layers. The layer markings were transferred onto the corresponding area of the serial Aβ immunohistochemically stained section using multiple reference points.

### 2.4 Areal fraction analysis

Aβ immunohistochemically stained sections with layer overlay were analysed per cortical layer for percentage area stained (Aβ load). A 6 mm width section along the gyrus was analysed for all cases, although differences in layer depth existed between cases. For each layer and each case, the same volumetric area was analysed. Images were opened in ImageJ (39) to select individual cortical layers. For each layer, 15 randomised regions of interest were generated using Python (Python 3.6.0, Python Software Foundation) for areal fraction analysis. A pre‐defined macro was used in ImageJ for threshold analysis. For each layer, the mean value from the 15 randomised regions of area stained was generated.

### 2.5 Aβ plaque‐type assessment

Evaluation was conducted blinded to mutation or clinical information. Aβ pathology was evaluated semi‐quantitatively based on a four‐point grading scale of 0–3 (none, sparse, moderate and frequent). Cortical tissue was assessed at 20x magnification within the same region as areal fraction analysis, to generate scores. We used the grading scale to assess Aβ plaque frequency for each cortical layer (1–6) to produce a layer‐specific score. These scores were then combined to generate an overall total Aβ plaque score for each case. Additionally, individual plaque types (diffuse, cored or CWP – identified as large circular diffuse Aβ plaques with defined edges) were assessed by layer (1–6) using the same grading system and those scores combined to generate a total score for each plaque type. Subpial Aβ pathology was also assessed on the 0–3 scale.

### 2.6 Cortical and leptomeningeal vessel counts

Aβ‐positive cortical and leptomeningeal CAA pathology was determined from IHC Aβ sections. Cortical CAA was also assessed across the six layers using a similar 0–3 grading scale and a combined total generated, as previously described (40, 41, 42). The proportion of vessels affected by Aβ deposition was also analysed. Brains donated to QSBB are not stripped of the leptomeninges, and leptomeninges were visible and present in all cases. Starting in the region selected for areal fraction analysis, 100 cortical vessels were counted, and the proportion of affected vessels were calculated as a percentage. Up to 100 leptomeningeal vessels were counted along the meninges. Only vessels cut cross‐sectionally showing the full circumference of the vessel wall were included in the analysis.

### 2.7 Statistical analysis

Analyses were performed using all cases both for the whole cohort and by sub‐groups based on mutation (including location where relevant) or *APOE4* status. The three mutation sub‐groups were *PSEN1* cases with a mutation before codon 200, *PSEN1* cases with a mutation after codon 200, and *APP* mutation cases. To classify *APOE4* status, cases were separated into those without an ε4 allele, for example ε2/3 and ε3/3, and those with at least one ε4 allele, for example ε3/4 and ε4/4. For analyses incorporating *APOE4* status 19 cases were used, as DNA was not available for case 9. Statistical analyses were performed using STATA 15.1 (StataCorp. 2017. Stata Statistical Software: Release 15. College Station, TX: StataCorp LLC).

#### 2.7.1 Clinical and genetic comparisons

Linear regression models compared both age at onset and disease duration by: (i) mutation sub‐group (adjusting for sex and *APOE4* status); (ii) sex (adjusting for mutation sub‐group and *APOE4* status); and (iii) *APOE4* status (adjusting for mutation sub‐group and sex).

#### 2.7.2 Aβ pathology scores

Associations between each of the four Aβ pathology scores (subpial, cored plaques, diffuse plaques, CWPs) and the two CAA scores (cortical CAA, leptomeningeal CAA) and (i) age at onset, and (ii) disease duration were investigated in the whole cohort using linear regression, adjusting for *APOE4* status. Each score was assessed for evidence of differences between mutation sub‐groups (Kruskal–Wallis) and by *APOE4* status (exact Mann–Whitney–Wilcoxon rank sum). All scores were then correlated pairwise against each other in the whole cohort and then in the sub‐groups in order to look for any patterns in the correlations (Kendall's tau‐b correlation coefficient, with statistical significance level determined using tables of critical values because of the small sample size). Finally, we compared individual layer Aβ pathology scores between sub‐groups (Kruskal–Wallis with Dunn's test for pairwise comparison between sub‐groups).

#### 2.7.3 Aβ load in the cortical tissue

Associations between mean total Aβ load (measured as a percentage area stained) across the six cortical layers and (i) age at onset and (ii) disease duration were assessed using a linear regression, adjusting for *APOE*4 status, in the whole cohort and in sub‐groups; these analyses were repeated for each of the six cortical layers individually. Mean Aβ load was compared between mutation sub‐group (Kruskal–Wallis with Dunn's test where appropriate) and by *APOE4* status (exact Mann–Whitney–Wilcoxon rank sum). Correlations between Aβ load and the frequency of the four different Aβ plaque pathologies and CAA scores were investigated in the whole cohort and in sub‐groups in order to look for any patterns in the correlations (Kendall's tau‐b as above). Finally, we assessed whether Aβ load differed between the different layers in the whole cohort (Friedman ANOVA). The Aβ load of each individual layer was then compared between the mutation sub‐groups, adjusted for *APOE4* status, using linear regression.

#### 2.7.4 Proportion of vessels affected by cortical CAA and leptomeningeal CAA

Linear regression models investigated whether in the whole cohort the proportions of vessels with CAA in the cortex and with CAA in the leptomeninges were associated with (i) age at onset and (ii) disease duration, adjusted for *APOE4* status. The proportions of CAA affected vessels were then compared between mutation sub‐groups (Kruskal–Wallis and Dunn's test) and by *APOE4* status (exact Mann–Whitney–Wilcoxon rank sum). Finally, we assessed whether the proportions of CAA affected vessels correlated with each of the four Aβ plaque pathologies scores and Aβ load (Kendall's tau‐b as above).

## 3 RESULTS

### 3.1 Neuropathological summary

All cases reached end‐stage AD with a score of A3B3C3 according to the current diagnostic criteria, indicating frequent neuritic plaques, neurofibrillary pathology spread to the occipital cortex reaching Braak and Braak stage 6 (except case 16 who scored 5) and Aβ plaque pathology in the cerebellum reaching Thal stage 5.

### 3.2 Clinical and genetic details

Subject details are summarised in Table 1. The 20 individuals with FAD in this study included: 16 subjects with *PSEN1* mutations, of which 10 were located pre‐codon 200 (four intron 4, one E120K, one S132A, one M139V, one M146I and two E184D) and six were located post‐codon 200 (one I202F, one L250S, two R278I, one E280G and one double mutation A434T & T291A), and four subjects with *APP* mutations (one V717L and three V717I). There were no statistically significant differences between the *APP* and the two *PSEN1* mutation sub‐groups for sex or *APOE4* status (Table 1).

**TABLE 1:** Study participants

Case	Sex	Age at onset (years)	Disease duration (years)^b^	Mutation	*APOE*	Braak Tau	Thal phase	CERAD	Alpha‐syn pathology	TDP−43 pathology	PMD (h/min)
1	F	36	5	PSEN1 Intron4	3/3	6	5	Frequent	None	None	16:15
2	F	35	16.9	PSEN1 Intron4	4/4	6	5	Frequent	Amygdala	Amygdala	32:30
3	F	39	8.1	PSEN1 Intron4	3/3	6	5	Frequent	None	Amygdala	–
4	M	42	9	PSEN1 Intron4	3/3	6	5	Frequent	Amygdala	Amygdala	43:10
5	F	31	6	PSEN1 E120K	3/3	6	5	Frequent	Amygdala	None	24:15
6	M	59	11	PSEN1 S132A	3/4	6	5	Frequent	Neocortical	None	161:15
7	F	41	8.9	PSEN1 M139V	3/3	6	5	Frequent	Amygdala	None	–
8	M	40	6	PSEN1 M146I	2/3	6	5	Frequent	Amygdala	None	115:35
9	F	40	13.6	PSEN1 E184D	‐	6	5	Frequent	None	None	153:30
10	F	45	13	PSEN1 E184D	3/4	6	5	Frequent	Amygdala	None	63:25
11	F	48	11	PSEN1 I202F	4/4	6	5	Frequent	Amygdala	None	26:15
12	M	47	11	PSEN1 L250S	3/3	6	5	Frequent	None	None	32:30
13	F	46	19	PSEN1 R278I	3/4	6	5	Frequent	Amygdala	None	31:55
14	M	54	12	PSEN1 R278I	2/3	6	5	Frequent	Amygdala	Amygdala	77:45
15	F	42	11	PSEN1 E280G	3/4	6	5	Frequent	Amygdala	None	11:00
16	M	42	5	PSEN1 A434T & T291A	3/3	5	5	Frequent	None	None	43:50
17	F	51	8.7	APP V717L	3/3	6	5	Frequent	None	None	89:42
18	M	56	10.1	APP V717I	3/3	6	5	Frequent	Amygdala	None	68:05
19	F	50	6.5	APP V717I	3/3	6	5	Frequent	Amygdala	None	16:25
20	M	49	13	APP V717I	4/4	6	5	Frequent	Amygdala	None	32:10
Mean	8M:12F	45.05	10.24								
	**Whole cohort N = 20**	***PSEN1* pre‐codon 200 N = 10**	***PSEN1* post‐codon 200 N = 6**	***APP* N = 4**	***p*‐value** *						
*APOE* (% *APOE4* carrier^a^)	37	33	50	25	0.70						
Sex (% female)	60	70	50	50	0.60						

Note

‘–’represents missing data. No *APOE* data for case 9.

Abbreviations: F, female; M, male; PMD, post mortem delay.

^a^ N = 19 as *PSEN1* pre‐codon 200 case 9 had no *APOE* genotype data available.

^b^ Disease duration accuracy based on available data.

* Fisher exact test.

Regression analyses for age at onset and disease duration were conducted, with adjustments for mutation group, sex and *APOE*4 status, where relevant (Table 2). Case 9 has no *APOE* genotype data available so was not included in this analysis. The mean age at onset of the whole cohort, was lower in the *PSEN1* pre‐codon 200 group (41.6 years) than the *PSEN1* post‐codon 200 group (46.5 years) and the *APP* group (51.5 years). After adjusting for sex and *APOE4* status, there was evidence of an association between age at onset and mutation sub‐group, with mean age at onset an estimated 8.8 years older (95% CI: 1.5, 16.1; *p* = 0.02) for *APP* compared with *PSEN1* pre‐codon 200 mutation carriers. There were no other statistically significant associations between mutation sub‐group and age at onset or disease duration.

**TABLE 2:** Clinical comparisons

	Observed mean (years) (N = 20)	Model coefficient (years) (N = 19)	95% CI	*p*‐value
*Age at onset*				
Mutation group^a^				*p* = 0.06
*APP* vs *PSEN1* pre‐codon 200^a^	51.5 vs 41.6	8.8	1.5, 16.1	*p* = 0.02*
*APP* vs *PSEN1* post‐codon 200^a^	51.5 vs 46.5	5.9	−2.0, 13.8	*p* = 0.13
*PSEN1* pre‐codon 200 vs *PSEN1* post‐codon 200^a^	41.8 vs 46.5	−2.9	−9.4, 3.6	*p* = 0.35
Females vs males^b^	42.0 vs 49.6	−7.3	−13.1, −1.4	*p* = 0.02*
*APOE*4 carrier vs non‐carrier^c^	46.3 vs 44.8	3.5	−2.5, 9.5	*p* = 0.23
*Disease duration*				
Mutation group^a^				*p* = 0.73
*APP* vs *PSEN1* pre‐codon 200^a^	9.6 vs 9.8	0.7	−3.1, 4.4	*p* = 0.71
*APP* vs *PSEN1* post‐codon 200^a^	9.6 vs 11.5	−0.6	−4.6, 3.5	*p* = 0.77
*PSEN1* pre‐codon 200 vs *PSEN1* post‐codon 200^a^	9.3 vs 11.5	−1.2	−4.6, 2.1	*p* = 0.44
Females vs males^b^	10.6 vs 9.6	−0.2	−3.2, 2.8	*p* = 0.89
*APOE*4 carrier vs non‐carrier^c^	13.6 vs 8.0	5.4	2.4, 8.5	*p* = 0.002**

Note

Observed mean for whole cohort (N = 20), except for *APOE* where N = 19. Linear regression models compared age at onset and disease duration by mutation subgroup, sex and *APOE4* status (N = 19). Asterisks represent statistically significant correlations.

^a^ Adjusted for sex and *APOE* genotype.

^b^ Adjusted for mutation sub‐group and *APOE* genotype.

^c^ Adjusted for mutation sub‐group and sex.

* *p* < 0.05

** *p* < 0.01.

In the cohort as a whole, females had a lower observed mean age at onset than males (42.0 years vs 49.6 years). After adjusting for mutation sub‐group and *APOE4* status, mean age at onset was an estimated 7.3 years younger (95% CI: 13.1, 1.4) for females compared with males (*p* = 0.02). No evidence of an association between disease duration and sex was found (Table 2). No statistically significant association between *APOE4* status and mean age at onset was found. However, there was strong evidence of an association between disease duration and *APOE4* status, after adjusting for mutation sub‐group and sex, with mean disease duration an estimated 5.4 years longer (95% CI: 2.4, 8.5) for individuals with the *APOE4* allele, compared with those without (*p* = 0.002) (Table 2).

### 3.3 Aβ pathology scores

#### 3.3.1 Associations with clinical data

**FIGURE 1:**
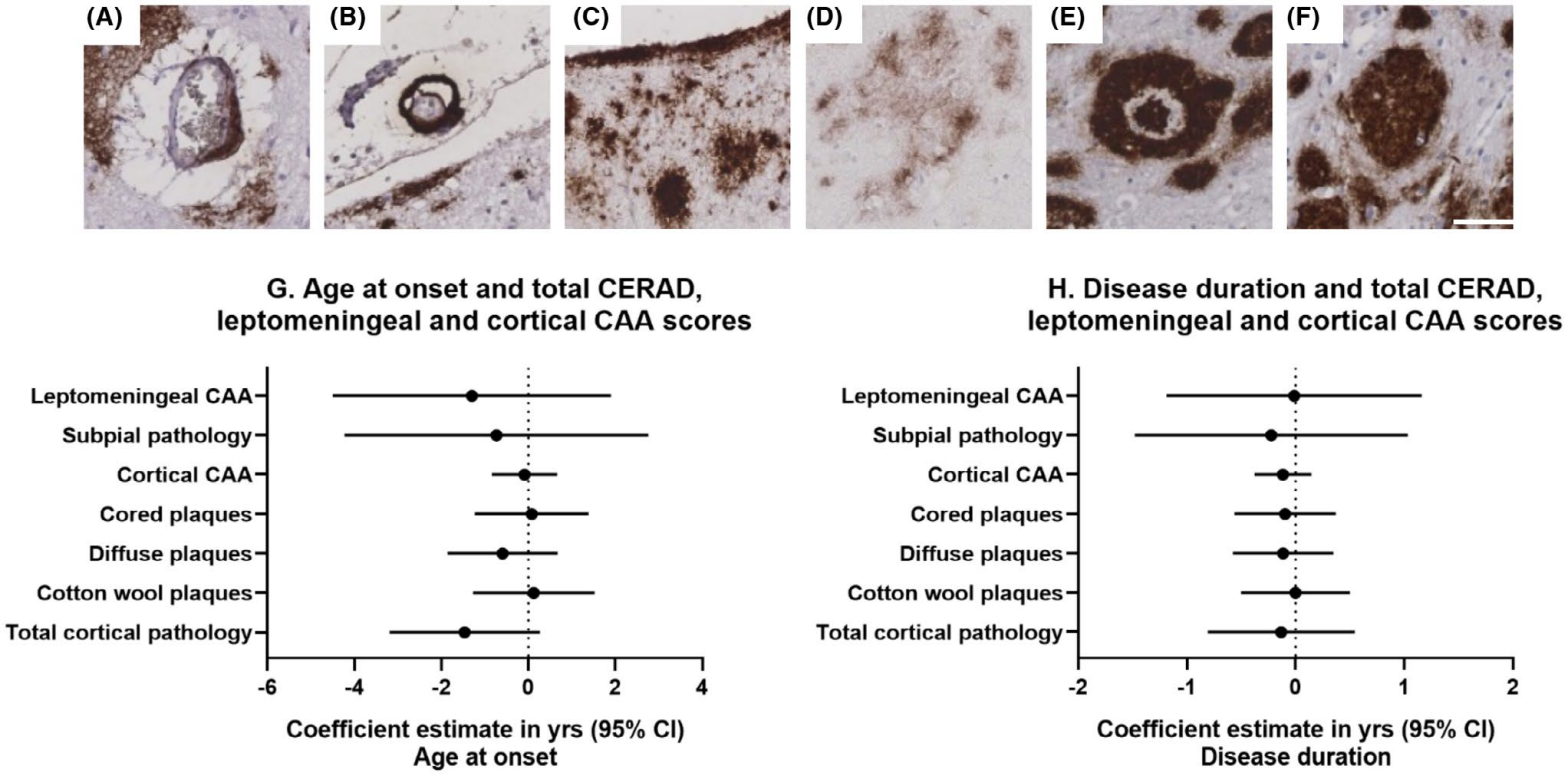
Representative images of cortical and leptomeningeal CAA (A & B), subpial pathology (C) and of Aβ plaque types (D: diffuse, E: Cored, F: CWP). White scale bar =50um, 200x objective. Linear regression adjusted for *APOE4* status showing the association between total Aβ pathology scores, leptomeningeal CAA, cortical CAA and age at onset (G) and disease duration (H). Coefficient estimates and 95% confidence intervals for the whole cohort are represented for each total Aβ pathology score and the leptomeningeal and cortical CAA scores.

The frequency of the total Aβ pathology types (representative images shown in Figure 1A–F) were analysed in relation to age at onset and disease duration. In the whole cohort, there were negative associations between leptomeningeal CAA, Aβ pathology scores (subpial pathology, diffuse plaques and total cortical pathology) and age at onset, while cortical CAA, cored plaques and CWP had little association. Negative associations between cortical CAA, Aβ pathology scores (cored and diffuse plaques and total cortical pathology) and disease duration was also seen. However, none of the adjusted associations were statistically significant, with small effect sizes and large CIs (p‐values ranged from *p* = 0.09 to *p* = 1.00, Figure 1G,H).

#### 3.3.2 Associations by mutation sub‐groups

**FIGURE 2:**
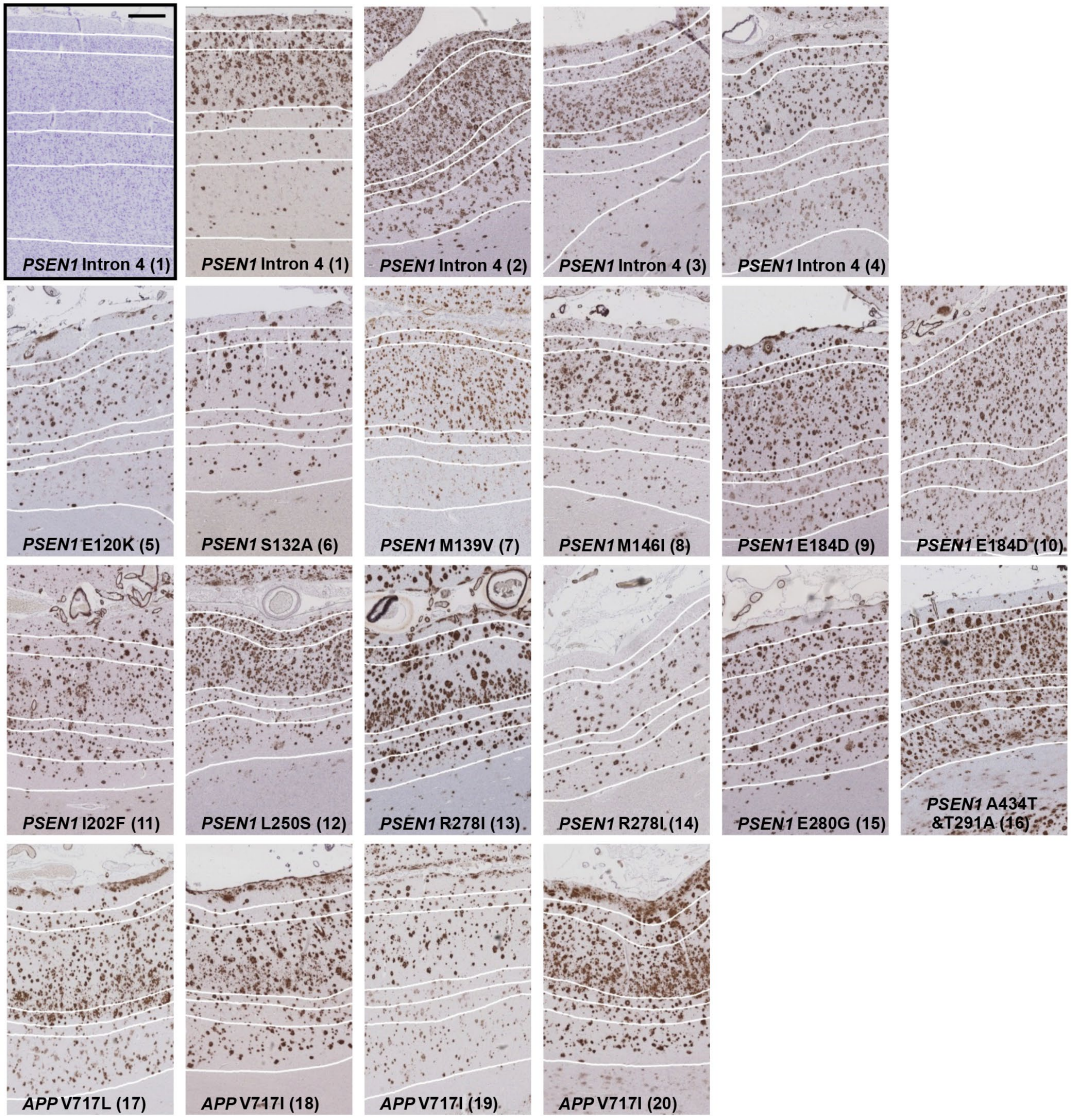
Representative images of Aβ immunohistochemically stained frontal cortex of each individual case within the mutation sub‐groups, *PSEN1* pre‐codon 200 (10 cases), *PSEN1* post‐codon 200 (6 cases) and *APP* (4 cases). One Nissl stained *PSEN1* Intron 4 mutation tissue section is shown (black box) to highlight how the layers were defined based on cellular morphology and applied to the serial Aβ section. The 6 cortical layers are defined by white lines, with layer 1 at the pial surface and layer 6 adjacent to the white matter. Black scale bar =500µm. Numbers in brackets refer to case number.

Microscopically, large heterogeneity in the appearance of Aβ pathology was evident throughout the cortical layers of the FAD cases (Figure 2). Table 3 shows the Aβ pathology scores for each case, giving the leptomeningeal CAA and subpial Aβ pathology, cortical CAA and plaque types in each cortical layer and the overall levels of Aβ plaque deposition. Observed leptomeningeal CAA and subpial Aβ pathology differed markedly between cases, being either absent, sparse, moderate or frequent. Based on microscopic visual inspection of the Aβ distribution throughout the cortical layers, variations were also observed between carriers of the same mutation. For example, one *PSEN1* R278I mutation case (case 13) contained markedly more Aβ pathology compared with the other *PSEN1* R278I case (case 14); interestingly case 13 had the genotype *APOE3*/*4* compared to case 14 which is *APOE2*/*3* (Figure 2). Additionally, differences within the pattern and distribution of plaques across the layers could be seen between these two cases, with case 13 having larger and greater plaque deposition particularly in layer 3, and greater cortical CAA and subpial deposition (Figure 2, Table 3E,A, and the subpial pathology row). However, similarities in layer deposition could be seen in other carriers with the same mutation, such as the *PSEN1* E184D carriers, with denser deposition of Aβ across all layers (cases 9 and 10, Figure 2). Similarly, distinct distribution patterns could be seen across different cases, with *APP* V717L case 17 and V717I cases 18 and 20 all showing frequent subpial Aβ deposition and distinct plaque gradient from the upper to the lower of layer 3 (Figure 2). In contrast, *PSEN1* E120K and S132A (cases 5 and 6) appear to have a more uniform distribution across layer 3 (Figure 2). Most cases showed the highest amount of Aβ staining within cortical layer 3, while the amount in the lower layers varied e.g. *PSEN1* M139V (case 7) has very little lower layer deposition compared to the dense lower layer deposition seen in *PSEN1* A434T & T291A (case 16) (Figure 2, Table 3B,D,E).

**TABLE 3:**
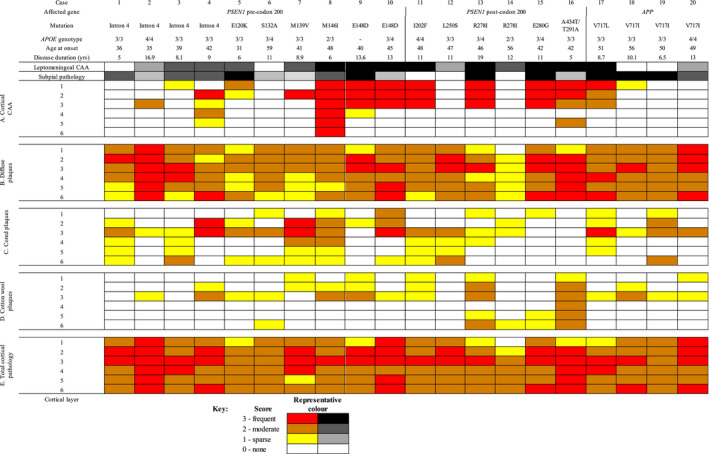
Tables showing total score of Aβ pathology type and leptomeningeal and cortical CAA per cortical layer by individual case

Note

Leptomeningeal and subpial pathology varies between cases, with presence of one not indicative of the other. (A) CAA pathology scores highlight a predominance of CAA in a subset of cases, with an upper layer predisposition. (B) Diffuse type plaques are seen throughout all cases to varying degrees between cases and within individual cases. (C&D) Cored and CWP pathology scores show that there is variation in presentation between cases, and within the same cases. (E) Total cortical pathology score shows that not all cases have the same level of plaque deposition. Dashed lines divide the sub‐groups, *PSEN1* pre‐codon 200, *PSEN1* post‐codon 200 and *APP*.

Microscopically, visual inspection of the slides revealed the *PSEN1* pre‐codon 200 and *APP* groups were more likely to have subpial pathology (presence in 100% of cases) than the *PSEN1* post‐codon 200 group (presence in 50% of cases). However, comparing each total Aβ pathology score in turn formally between the three mutation sub‐groups, there were no statistically significant differences in plaque pathology (subpial *p* = 0.10, cored *p* = 0.27, diffuse *p* = 0.46, CWP *p* = 0.62 and total cortical pathology *p* = 0.87, global tests Kruskal–Wallis), or CAA pathology (leptomeningeal CAA *p* = 0.17, cortical CAA *p* = 0.41). None of the Aβ pathologies differed by *APOE4* status.

#### 3.3.3 Correlations between Aβ pathology scores

**FIGURE 3:**
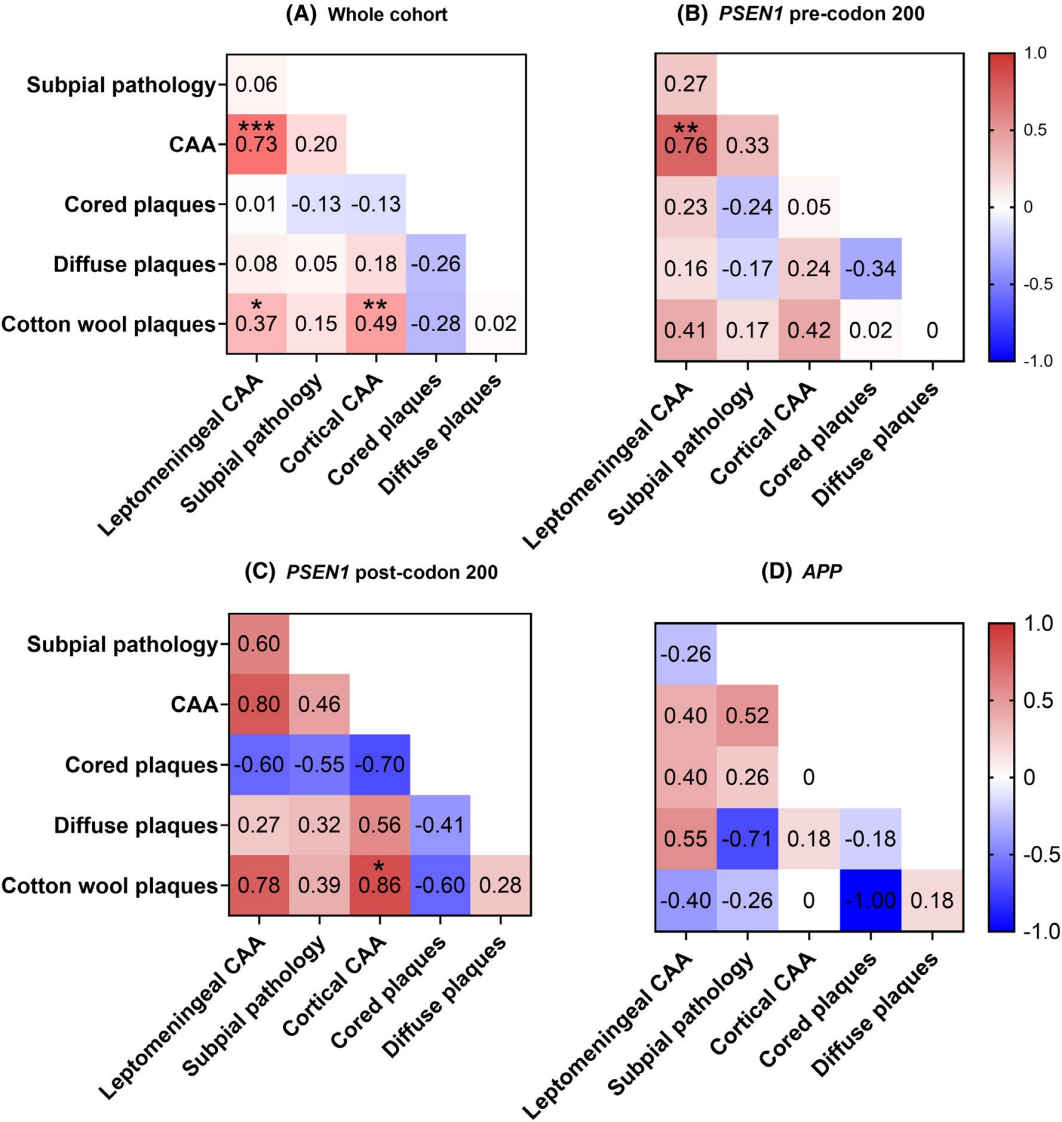
Correlation between total Aβ pathologies, leptomeningeal and cortical CAA: heat maps showing the relationship between the total Aβ pathology scores, leptomeningeal and cortical CAA ‐ A) Whole cohort, B) *PSEN1* pre‐codon 200, C) *PSEN1* post‐codon 200, D) *APP*. *τ*_b_ values for Kendall's tau‐b correlation coefficients are shown. Asterisks represent statistically significant correlations: *p < 0.05, **p < 0.01, ***p < 0.001. Red =positive correlation. Blue = negative correlation

In the whole cohort, the frequency of leptomeningeal CAA was correlated with total cortical CAA (correlation coefficient *τ*_b_ = 0.73 *p* ≤ 0.001, Kendall's tau‐b) (Figure 3A). When the sub‐groups were assessed separately there was a consistent positive correlation (Figure 3B–D) that remained in the two *PSEN1* sub‐groups. A trend for positive correlation between both total cortical and leptomeningeal CAA with total CWP score was seen across all groups except the small *APP* group. Specifically, in the whole cohort, cortical and leptomeningeal CAA scores were both positively correlated to total CWP scores (*τ*_b_ = 0.49 *p* < 0.01 and *τ*_b_ = 0.37 *p* < 0.05, respectively) and in the sub‐group analyses this correlation was particularly evident in the *PSEN1* post‐codon 200 group for cortical CAA (*τ*_b_ = 0.86, *p* < 0.05) and leptomeningeal CAA (*τ*_b_ = 0.77). In contrast, there was an observed trend for negative correlations between total cored plaque score and Aβ pathologies (subpial pathology, diffuse plaques, CWPs); this was particularly evident in the *PSEN1* post‐codon 200 group, and slightly evident for diffuse plaques and CWPs in the *APP* group (Figure 3A–D).

#### 3.3.4 Differences across cortical layers

As reported above, we did not find evidence of total Aβ pathology scores differing between the sub‐groups. However, when comparing individual cortical layer scores (0–3) between sub‐groups we found CWPs in layer 5 and 6 were only seen to any measurable extent in the *PSEN1* post‐codon 200 group (*p* = 0.02 and *p* = 0.02, respectively, Kruskal–Wallis). Specifically, in layer 5 the *PSEN1* post‐codon 200 group had a higher CWP score (0.67 ± 0.82) compared to the *PSEN1* pre‐codon 200 group (0 ± 0; *p* = 0.01, Dunn's test) and the *APP* group (0 ± 0; *p* = 0.04). This pattern was also seen in layer 6, with the *PSEN1* post‐codon 200 group again having a higher frequency of CWPs (1.0 ± 0.89) than the *PSEN*1 pre‐codon 200 (0.1 ± 0.32; *p* = 0.01, Dunn's test) and *APP* group (0.0 ± 0.0; *p* = 0.02, Dunn's test). There was, however, considerable layer 5 and 6 variability within the sub‐groups. No differences for other plaque types were found. There were also no patterns of differences at the layer level in total Aβ pathology scores between *APOE4* carriers and non‐carriers.

### 3.4 Aβ load in the cortical tissue

#### 3.4.1 Associations with clinical data

**FIGURE 4:**
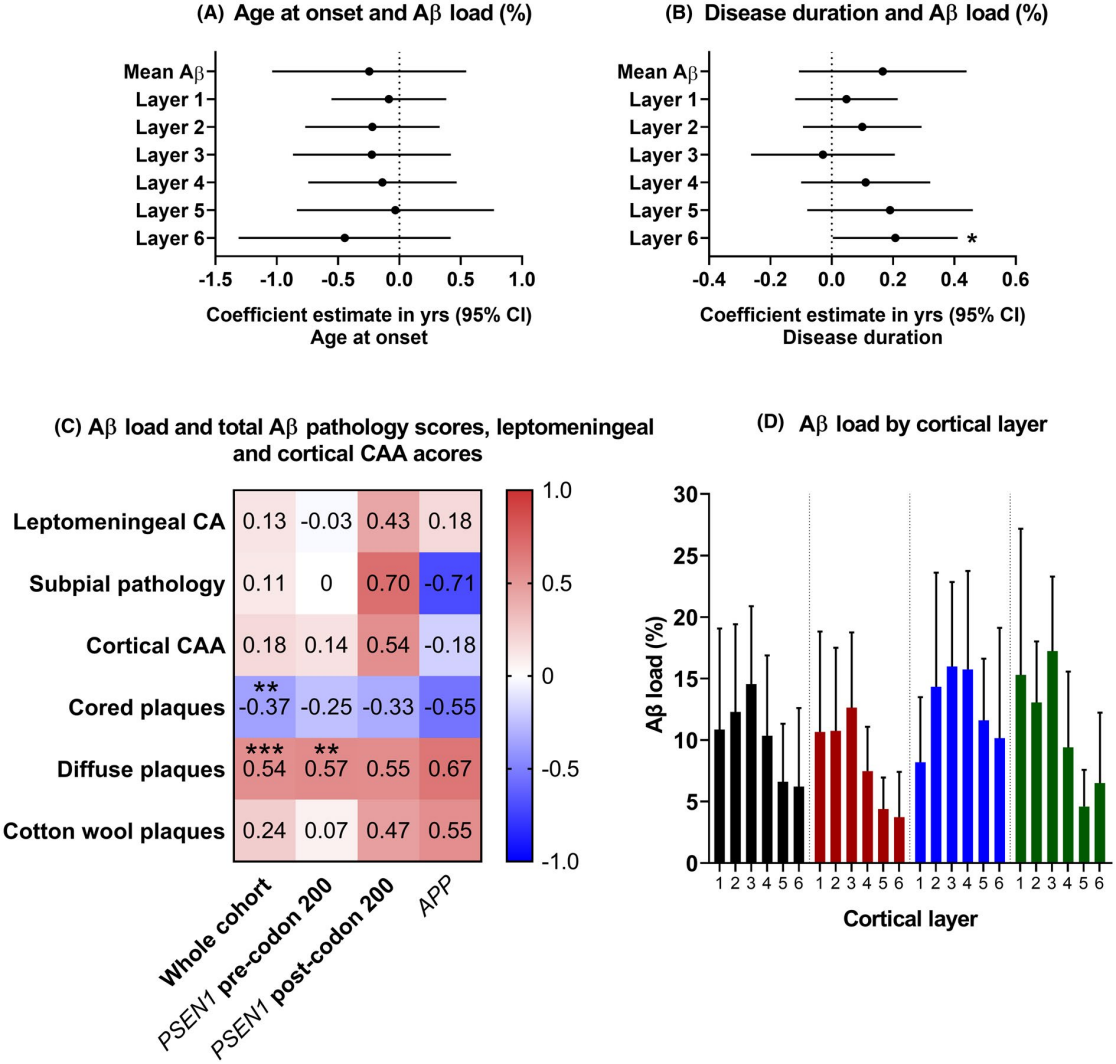
Linear regression adjusted for *APOE4* status showing the association between Aβ load and age at onset (A) and disease duration (B). Coefficient estimates and 95% confidence intervals for the whole cohort are represented, for the mean Aβ load and for each cortical layer. The coefficients are the estimated difference in the outcome (age at onset or disease duration) for a one percentage point increase in the explanatory variable, after adjusting for *APOE4* status. *p = 0.05. (C) Correlations between Aβ load and total Aβ pathology scores/ leptomeningeal and cortical CAA for the whole cohort and individual mutation sub‐groups. *τ*_b_ values for Kendall's tau‐b correlation coefficients are shown. Asterisks represent statistically significant correlations: *p < 0.05, **p < 0.01, ***p < 0.001. Red = positive correlation. Blue = negative correlation. (D) Graphical display of the mean Aβ load (%) per cortical layer for the whole cohort and individual mutation sub‐groups. Bars and error bars represent mean and SD

Associations between mean Aβ load (measured as a percentage area stained) across the six cortical layers and age at onset and disease duration are shown in Figure 4A,B. Mean Aβ load was not statistically significantly associated with age at onset although a consistent negative association was found with mean Aβ load and Aβ load for individual cortical layers. Mean Aβ load was non‐significantly positively associated with disease duration, and this trend was seen for most cortical layers, with evidence for an association found (*p* = 0.0003) for layer 6 Aβ load and disease duration, (an estimated 0.21 additional years of duration for each percentage point increase in Aβ load; 95% CI: 0.004, 0.41; *p* = 0.05, after adjusting for *APOE*4 status).

#### 3.4.2 By mutation sub‐groups

When mean Aβ load was compared between mutation sub‐groups, the *PSEN1* post‐codon 200 group had the highest observed mean load, followed by the *APP* group, whilst the *PSEN1* pre‐codon 200 group had the lowest (12.7% ± 6.4, 11% ± 5.5, 8.3% ± 3.8, respectively), although there was no statistically significant difference between the groups (*p* = 0.26 Kruskal–Wallis). Mean Aβ load was observed higher in *APOE4* carriers than non‐carriers (13.1% ± 5.9, 8.5% ± 4.3, respectively), although with only weak evidence (*p* = 0.08, exact Mann–Whitney–Wilcoxon rank sum), due perhaps to the small sample size.

#### 3.4.3 Correlations with Aβ pathology scores

Associations between Aβ load and the frequency of the different Aβ pathologies were investigated. Leptomeningeal CAA, subpial Aβ and cortical CAA did not significantly correlate with Aβ load. When the three different plaque types were examined separately, there was a clear trend of a negative correlation between cored plaque score and Aβ load, both for the whole cohort and for all sub‐groups (Figure 4C), and this was statistically significant for the whole cohort (*τ*_b_ = −0.37, *p* < 0.01 Kendall's tau‐b). This contrasted with the consistently positive correlations seen between diffuse plaque score and Aβ load, and between CWP score and Aβ load; these associations were statistically significant for diffuse plaques in the whole cohort (*p* < 0.001) and in the *PSEN1* pre‐codon 200 group (*p* < 0.01), Figure 4C.

#### 3.4.4 Differences across cortical layers

Visual inspection of the cases revealed that Aβ deposition was not evenly distributed across the cortical layers (see Figure 2). In the whole cohort and within each mutation sub‐group, observed mean Aβ load was consistently numerically highest in layer 3 compared to the remaining layers (whole cohort 14.57% ± 6.33; *PSEN1* pre‐codon 200 12.64% ± 6.12; *PSEN1* post‐codon 200 15.99% ± 6.88; *APP* 17.24% ± 6.07).

We assessed whether Aβ load differed between the different layers. There was evidence that this was the case for the whole cohort (*p* < 0.0001, Friedman ANOVA), with the highest load being observed in upper layers 2 and 3, a pattern that was also generally seen across the three sub‐groups (Figures 3 and 4D). The Aβ load of each individual layer was compared between the mutation sub‐groups, adjusted for *APOE4* status. We found evidence (*p* = 0.007) that Aβ load in layer 5 differed between sub‐groups, with mean Aβ load higher in the *PSEN1* post‐codon 200 group compared to both the *PSEN1* pre‐codon 200 group (7.16 percentage points higher; 95% CI: 2.9, 11.41; *p* = 0.003) and the *APP* group (6.91 percentage points higher; 95% CI: 1.68, 12.15; *p* = 0.01), after adjusting for *APOE4* status. There was weaker evidence (*p* = 0.06) for differences in Aβ load in layer 4, with *PSEN1* post‐codon 200 cases again having higher load than the *PSEN1* pre‐codon 200 group (7.99 percentage points higher; 95% CI: 1.51, 14.46; *p* = 0.02). No significant differences were found for the other layers. These data suggest that mutation location may be associated with the distribution of pathology across some cortical layers but larger sub‐group sizes would be needed to investigate this further.

### 3.5 Proportion of cortical and leptomeningeal vessels affected by CAA

In our cohort, vessels positive for CAA were observed in the frontal cortices of the following *PSEN1* mutations (three intron 4 mutation cases (1–4), E120K, M146I, both E148D, both E280G, L250S, both R278I, double mutation A434T &T291A). CAA was also present in the *APP* V717L and two *APP* V717I cases. Three cases (case 1: *PSEN1* intron 4, 6: *PSEN1 S132A* and 19: *APP* V717I) had no CAA present in the frontal cortices.

#### 3.5.1 Associations with clinical data

We assessed associations between the proportions of vessels with CAA in the cortex and leptomeninges and age at onset or disease duration. In the whole cohort, there was a trend for both older age at onset and longer disease duration to be associated with a smaller proportion of vessels with CAA, however these associations were not statistically significant (Figure S1).

#### 3.5.2 Differences between sub‐groups

**FIGURE 5:**
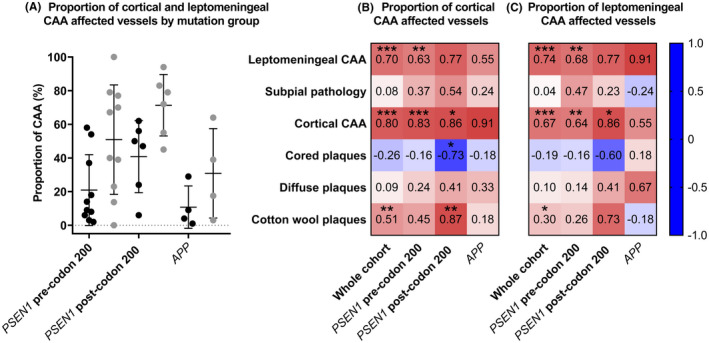
(A) Comparison of the proportion of cortical and leptomeningeal CAA between mutation groups. *PSEN1* post‐codon 200 cases have significantly higher cortical and leptomeningeal CAA compared to the *APP* cases (*p* = 0.02 and *p* = 0.01, Dunn's test), with near significant higher proportion of cortical and leptomeningeal CAA compared to the *PSEN1* post‐codon 200 group (*p* = 0.06 and *p* = 0.07). Bars represent mean and SD. Correlations between the proportion of CAA affected vessels in the cortex (B) and leptomeninges (C) and the total Aβ pathology, leptomeningeal and cortical CAA scores for the whole cohort and mutation sub‐groups. Kendall's tau‐b *τ*_b_ correlation coefficients are shown. Red = positive correlation. Blue = negative correlation. Asterisks represent statistically significant correlations: *p < 0.05, **p < 0.01, ***p < 0.001

The proportion of CAA affected vessels was compared between mutation sub‐groups. *PSEN1* post‐codon 200 cases had the highest observed proportion of affected vessels in both the cortex and leptomeninges compared to the other groups, with all subjects in the *PSEN1* post‐codon 200 mutation sub‐group demonstrating amyloid‐beta deposition in at least 6% of their cortical vessels and 45% of their leptomeningeal vessels compared to a minimum of 2% and 0% in the *PSEN1* pre‐codon 200 group and 1% and 3% in the *APP* group, respectively. While there was only very weak evidence for differences between the sub‐groups (*p* = 0.10 and *p* = 0.08, Kruskal–Wallis), this may have been because of small sub‐group sizes so we investigated pairwise comparisons. The proportion of vessels with cortical CAA was non‐statistically higher (*p* = 0.06, Dunn's test) in the *PSEN1* post‐codon 200 (40.83% ± 21.43) compared to the *PSEN1* pre‐codon 200 group (16.78% ± 17.56), and statistically higher (*p* = 0.02) compared to the *APP* group (10.75% ± 12.61). Additionally, the proportion of vessels with leptomeningeal CAA was higher (*p* = 0.07) in the *PSEN1* post‐codon 200 group (71.33% ± 18.27) compared to the *PSEN1* pre‐codon 200 group (45.43% ± 29.24), and higher (*p* = 0.01) than the *APP* group (30.88% ± 26.58) (Figure 5A). There were no differences in the proportions of cortical and leptomeningeal CAA by *APOE4* status (*p* = 0.31, *p* = 0.47, exact Mann–Whitney–Wilcoxon rank sum).

#### 3.5.3 Correlations with Aβ pathologies

We assessed whether the proportions of cortical and leptomeningeal CAA affected vessels correlated with the total Aβ pathology scores. In contrast to the pattern seen for all other Aβ pathology scores, we found a negative correlation between cored plaque scores and the proportions of both cortical and leptomeningeal CAA affected vessels. While these relationships did not reach statistical significance, the negative correlations were seen consistently in the whole cohort, in all sub‐groups for cortical CAA, and in all but the small *APP* sub‐group for leptomeningeal CAA (see Figure 5B,C for the correlation coefficients). All other total Aβ pathology scores were consistently positively correlated with the proportions of both cortical and leptomeningeal CAA affected vessels in the whole cohort and also in all the sub‐groups, except again in the small *APP* group. As expected CAA scores and the proportions of CAA vessels were positively correlated and in the whole cohort this was statistically significant (all *p* < 0.001) (Figure 5B,C). Of particular interest were the results for CWPs, where in the whole cohort there was evidence for a positive correlation with the proportion of cortical CAA (*τ*_b_ = 0.51, *p* ≤ 0.01, Kendall's tau‐b) and a consistent but weaker correlation with leptomeningeal CAA (*τ*_b_ = 0.30, *p* < 0.05). We also assessed if the mean Aβ load for the whole cohort was significantly associated with the proportions of cortical CAA or leptomeningeal CAA, with no statistically significant correlations found (*τ*_b_ = 0.17 and *τ*_b_ = 0.10, respectively).

## 4 DISCUSSION

In this study, we investigated associations between genotype, clinical data and Aβ pathology in a cohort of FAD cases. We found no differences in the presence of Aβ pathologies and *APOE* status, although the overall amount of Aβ was found to be higher in *APOE4* carriers than non‐carriers. In all mutation carriers, the highest amount of Aβ was found in layer 3. Aβ load also correlated with the amount of diffuse deposits found throughout the cortical layers rather than the neuritic plaques. It was also of interest that two cases with the same autosomal dominant mutations, R278I, displayed different patterns of Aβ deposition. Although one case carried an *APOE4* allele, suggesting this may play a role in modifying the deposition of Aβ. We found positive associations between the frequency of CWPs and both cortical and leptomeningeal CAA (total CAA scores and proportions of CAA affected vessels). Furthermore, the presence of CWPs was observed to be higher in lower cortical layers in some *PSEN1* post‐codon 200 cases, which may relate to observed clinical and pathological differences in FAD patients with mutations located post‐codon 200. In this study we used a pan‐Aβ antibody which stains all Aβ peptides and morphological conformations containing amino acid residues 8–17. It is clearly recognised within the literature that different isoforms and species are found in the different pathologies. However, in this study, we investigated the overall deposition of the Aβ peptides across the cortical layers rather than dissecting out the isoforms present in relation to the different structural forms of the peptides (soluble, oligomeric or amyloid). This could be an interesting direction for future work now that we have an overview of the total Aβ deposition.

In line with previous findings, our data show that mutation type is associated with age at onset in FAD. Specifically, *PSEN1* mutations located pre‐codon 200 had a significantly earlier age at onset than *APP* mutations. In our cohort, females had an earlier age at onset than males, after adjusting for mutation sub‐group and *APOE4* status. A trend for younger age at onset in females has also been observed in a meta‐analysis of FAD, although it did not reach statistical significance (9). Our findings highlight the importance of considering and investigating potential sex differences in disease manifestation and progression in FAD and the mechanisms that may be driving such differences. This is an area of growing interest in AD research (43), which could be informed by the insights gained from the study of FAD, where patients are typically young and lack the co‐morbidities that can confound studies of older individuals with dementia.

Similar to findings in other FAD studies, *APOE4* status in our cohort did not affect age at onset (9, 44). However, we found that possession of at least one ε4 allele was associated with longer disease duration, after adjusting for mutation sub‐group and sex. Our pathological analysis did not demonstrate evidence of significant differences in the type, frequency or layer distribution of Aβ pathology between *APOE4* carriers and non‐carriers, raising the question of whether the differences we observed in disease duration may be mediated by mechanisms beyond amyloid‐beta. A recent survival analysis from our centre (which included the individuals in the current study) suggested *PSEN1* mutation carriers with an *APOE4* allele may have longer survival (14). However, the effect of the *APOE4* genotype on disease duration in FAD is not well known and in sporadic AD its effect is uncertain. In a meta‐analysis including up to 1,700 sporadic AD cases, it was shown to have no effect (45), however, in two prior studies of AD patients, a trend towards increased disease duration in *APOE4* carriers was observed (46) and, separately, increased survival of *APOE4* carriers was found (47). Our finding that *APOE4* was associated with increased disease duration in this FAD cohort suggests that the impact of *APOE4* on disease progression in AD should be explored further in future larger studies.

In the whole cohort, total pathological Aβ plaques were not significantly associated with age at onset or disease duration. Similarly, Aβ load was not significantly associated with age at onset, although Aβ load in layer 6 was positively associated with disease duration and this trend was consistent for the remaining layers. However, the associations require verifying in a larger cohort. Although differing Aβ distribution between individual cases was evident macroscopically, Aβ load was consistently higher in layer 3 for all sub‐groups, as found in previous studies of sporadic and familial AD (25, 26, 27, 30). Various hypotheses suggest an increased vulnerability to AD pathomechanisms of certain neuronal populations (48, 49, 50, 51, 52). In particular, there is some evidence that cortical–cortical connections, predominant in layer 3, may be particularly vulnerable (49). Interestingly, it has been shown in murine models that layer 3 neurons are more susceptible to Aβ toxicity than layer 5–6 neurons, indicating that there may be some intrinsic factor involved (53). A range of earlier studies has carried out investigations into amyloidogenic tau pathology in AD cortical tissue (indicated by senile plaques, neuritic plaques or NFT’s), finding this to be observed with a predominance for cortical layer 3 and 5 (24, 25, 26, 27, 54) and highlighting preferential deposition and disruption in cortico‐corticol connective regions.

Despite there being no significant differences in the total Aβ pathology scores between groups, a significantly higher frequency of CWPs in layers 5 & 6 was observed in the *PSEN1* post‐codon 200 mutation carriers. This finding should be interpreted with some caution because of the small number of subjects. However, CWP pathology has often been seen in kindreds and individual cases with *PSEN1* mutations post‐codon 200, such as the *PSEN1* exon 9 deletion, G217R, P264L and L435F mutations (55, 56, 57, 58, 59). This suggests certain mutations post‐codon 200 support the development of CWPs; however, they can also be found in cases with *PSEN1* pre‐codon 200 mutations (60). The reasons why different types of Aβ deposit are formed, and the role that these various Aβ pathologies play in AD pathogenesis, are not yet fully understood. One possibility is that differences in production of Aβ peptides (e.g., Aβ40, 42, 43) play a role in influencing plaque deposition. Different FAD mutations cause distinct alterations in the spectrum of Aβ peptides produced (16) and these peptides vary in their potential for aggregation, which is likely to influence where they deposit and how they are involved in any associated vascular or inflammatory processes. For example, two of the individuals in the post‐codon 200 group in this study carried the R278I mutation. This, and other post‐codon 200 mutations, have been found to cause a particular increase in Aβ43 together with impairment of PSEN1 autoproteolysis (16, 61). It has been speculated that altered processing of substrates other than APP, at an endopeptidase level, may also contribute to variability in the effects of *PSEN1* mutations (3, 61) potentially representing an additional factor contributing to heterogeneity in Aβ pathology. Understanding the aetiology of CWPs is important because of their association with clinical phenotypes such as spastic paraparesis and other motor symptoms (31, 32, 62). Motor symptoms and spastic paraparesis are observed more frequently in *PSEN1* post‐codon 200 cases (3, 63, 64, 65, 66), supporting a link between the clinical phenotype and CWP pathology. The increased frequency of CWPs in lower layers, especially layer 5, that we observed in our *PSEN1* post‐codon 200 mutation cohort is perhaps consistent with this clinicopathological association as cortical layer 5 contains projections to the striatum, which is involved in motor control (67). While the evidence suggests a link, firm conclusions cannot be drawn as CWPs can exist without spastic paraparesis and CWPs can be found in sporadic AD, albeit infrequently (68, 69). Further investigation of the localisation of CWPs and their relationship with spastic paraparesis, particularly in *PSEN1* post‐codon 200 cases, may provide more detail on how these observations may be linked. In future studies with larger cohorts, it would be of interest to also examine the influence of *PSEN1* mutation location in more detail, moving beyond a simple codon 200 cut‐off to investigate the structural and functional consequences of mutations in different positions and how these relate to neuropathological features.

Analysis of the proportions of CAA revealed no significant correlations with Aβ load; however, positive correlations between CAA and CWPs were found in the full cohort and in the *PSEN1* post‐codon 200 group separately using both the scoring system and vessel count measures. Previously, CWPs have been observed in FAD cases with severe CAA (70) and CWPs in FAD have been found to correlate with white matter hyperintensities on MRI, which are a potential imaging marker of CAA (42). However, the precise link between these pathologies is unclear and interestingly CAA is predominantly composed of Aβ40 (71) while CWPs are composed mainly of Aβ42 (69, 72, 73, 74), although Aβ40 can be present (33). Despite this, our findings relating to CWPs and CAA, especially in reference to *PSEN1* post‐codon 200 mutations, supports a pathological link, which requires verification in studies with larger cohorts. Given further immunohistochemical investigations with peptide‐specific antibodies, we would expect to see predominance of Aβ40 in CAA and cored plaques, with greater abundance of Aβ42 in diffuse and CWP. The predisposition of Aβ40 to deposit in CAA or cored plaques may also account for the observed negative correlation between these two pathologies, wherein Aβ40 may be driven towards one of these main fates – depositing predominantly as either CAA or cored plaques.

In our cohort, cortical and leptomeningeal CAA were not correlated with age at onset or disease duration. There was a suggestion of differences between mutation sub‐groups but this would require larger sample sizes to investigate further. Interestingly, some of our cases had little to no CAA, suggesting it is not an inevitable consequence of Aβ pathology. Analysing a wider range of genetic cases could help decipher how different mutations are implicated in the development of CAA, for instance, because of effects on PSEN1 substrates other than APP (75) or on Aβ profiles and their aggregation potentials (11, 18). Despite the heterogeneity of CAA, understanding its role is important, especially as amyloid‐beta modifying therapies may lead to an increase in CAA and with adverse vascular events in AD, with *APOE* status influencing outcomes (76, 77, 78, 79).

All FAD cases in our cohort had been neuropathologically diagnosed with end‐stage severe AD. This study focussed on the distribution of Aβ throughout the cortical layers. The study did not focus on tau pathology as differences in tau deposition between cases would be difficult to observe using immunohistochemical analysis as the frontal cortex is full of neuropil threads. However, using a pan‐ Aβ immunohistochemical analysis binding the majority of Aβ species, we found that a more detailed investigation of the type and layer distribution of amyloid‐beta pathology highlighted differences between cases, with mutation location associated with certain clinical and pathological features. Broad pathological categorisations may mask more detailed mutation‐specific effects on pathogenic processes. Investigating these differences could provide better knowledge of the mechanisms of pathology and be an important tool for better understanding heterogeneity in AD. However, our cohort was small with only one case with certain mutations available. It could be misleading to generalise from one mutation case to all others, and it would be essential to have a larger number of cases to make true mutation‐based connections to pathological heterogeneity. Related to this, despite the distinct pathology patterns we observed between mutation sub‐groups, differences in pathology were also noted between cases with the same mutation indicating that other factors may influence pathological and clinical features in addition to the causative FAD mutations. We have highlighted some of those factors, with *APOE4* genotype status being of importance and associated with increased disease duration. Additionally, we observed sex differences in age at onset with a younger age at onset in females, although larger studies will be needed to confirm this finding. Further investigation of the role of these factors in FAD may provide insights into how they are involved in the mechanisms underlying AD in general.

## CONFLICT OF INTEREST

The authors have no conflict of interests.

## AUTHOR CONTRIBUTIONS

Tammaryn Lashley and Natalie S. Ryan conceptualised the study. Material preparation, data collection and analysis were performed by Nanet Willumsen, and statistical analysis by Teresa Poole and Jennifer M. Nicholas. The first draft of the manuscript was written by Nanet Willumsen and all authors commented on previous versions of the manuscript. All authors read and approved the final manuscript.

## ETHICAL APPROVAL

Ethical approval was obtained for the use of post‐mortem human brain tissue from the Local Research Ethics Committee of the National Hospital for Neurology and Neurosurgery. TL and NR drafted the study design, NW undertook the wet lab work and analysis. All authors read and contributed to the final manuscript.

## Supporting information


**FIGURE S1** Association between clinical data and the proportion of cortical and leptomeningeal CAA affected vessels

## Data Availability

The data that support the findings of this study are available from the corresponding author upon reasonable request.
