# Supplementary figures and images for: Variability in the type and layer distribution of cortical Aβ pathology in familial Alzheimer’s disease
Source: Brain Pathol. 2021 Jul 28;32(3):e13009. doi: 10.1111/bpa.13009 (PMC9048809; doi:10.1111/bpa.13009)

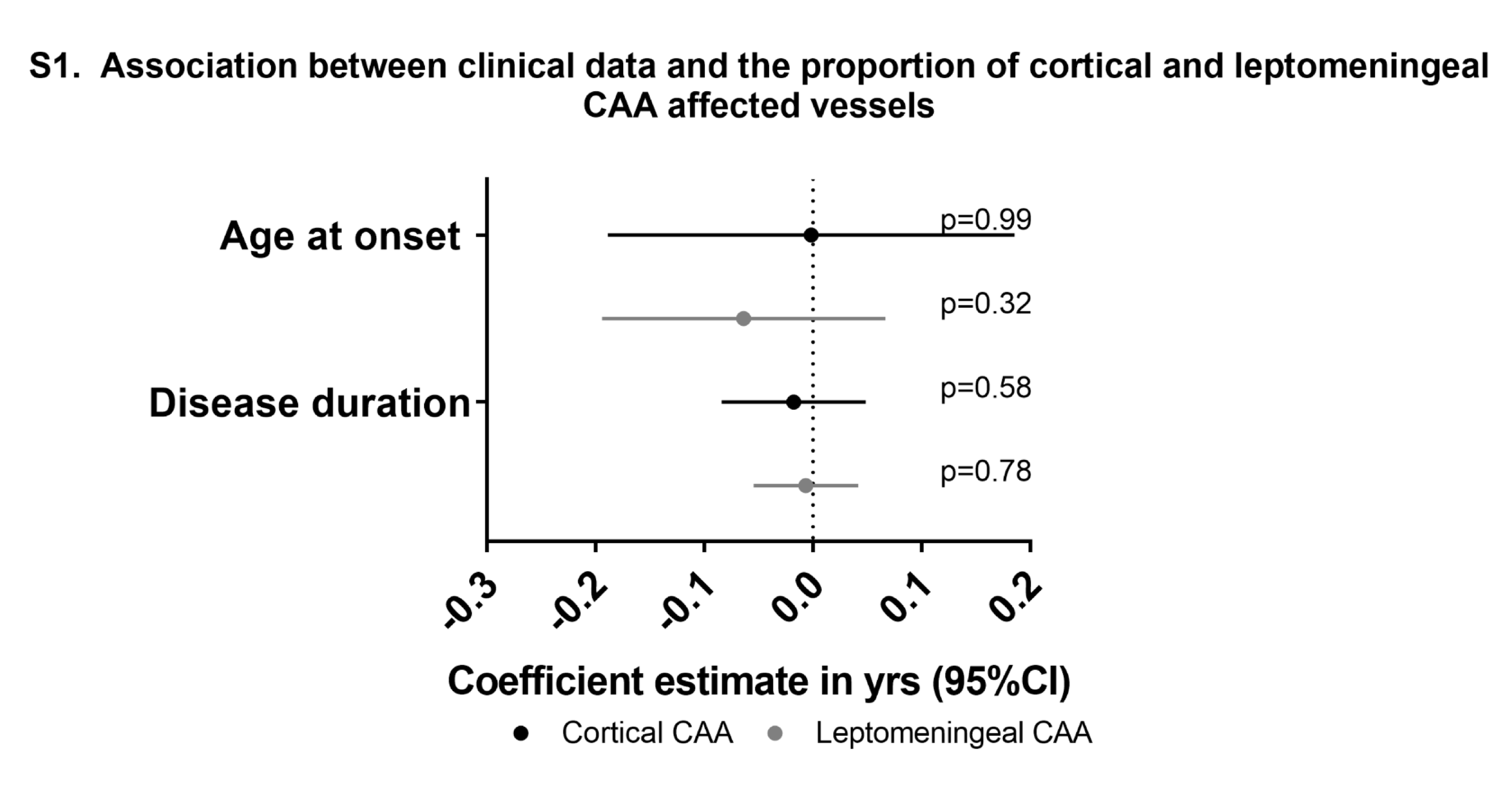

Supplement: Supplementary file 1 — FIGURE S1 Association between clinical data and the proportion of cortical and leptomeningeal CAA affected vessels [file BPA-32-e13009-s001.tif]
